# The Potential Role of ORM2 in the Development of Colorectal Cancer

**DOI:** 10.1371/journal.pone.0031868

**Published:** 2012-02-21

**Authors:** Xuhua Zhang, Zhiying Xiao, Xiaoyong Liu, Lutao Du, Lili Wang, Shun Wang, Ni Zheng, Guixi Zheng, Wei Li, Xin Zhang, Zhaogang Dong, Xuewei Zhuang, Chuanxin Wang

**Affiliations:** 1 Department of Clinical Laboratory, Qilu Hospital, Shandong University, Jinan, Shandong, China; 2 Department of Clinical Laboratory, The Second Hospital, Shandong University, Jinan, Shandong, China; 3 Department of Urology, The Second Hospital, Shandong University, Jinan, Shandong, China; 4 Department of Biology, McGill University, Montreal, Quebec, Canada; The Chinese University of Hong Kong, Hong Kong

## Abstract

Colorectal cancer (CRC) is the third most common malignancy in the world. The risk of death is closely correlated to the stage of CRC at the time of primary diagnosis. Therefore, there is a compelling need for the identification of blood biomarkers that can enable early detection of CRC. We used a quantitative proteomic approach with isobaric labeling (iTRAQ) to examine changes in the plasma proteome of 10 patients with CRC compared to healthy volunteers. Enzyme-Linked Immunosorbnent Assay (ELISA) and Western blot were used for further validation. In our quantitative proteomics analysis, we detected 75 human plasma proteins with more than 95% confidence using iTRAQ labeling in conjunction with microQ-TOF MS. 9 up-regulated and 4 down-regulated proteins were observed in the CRC group. The ORM2 level in plasma was confirmed to be significantly elevated in patients suffering from CRC compared with the controls. ORM2 expression in CRC tissues was significantly increased compared with that in corresponding adjacent normal mucous tissues (*P*<0.001). ITRAQ together with Q-TOF/MS is a sensitive and reproducible technique of quantitative proteomics. Alteration in expression of ORM2 suggests that ORM2 could be used as a potential biomarker in the diagnosis of CRC.

## Introduction

In 2009, the American Cancer Society estimated a total of 146,970 new colorectal cancer (CRC) cases and 40% deaths in the United States. CRC is expected to be the third most common cancer in America. Since 1975, there has been a notable improvement in relative CRC 5-year survival rates, as a result of earlier diagnosis and improving treatments [1]. Sigmoidoscopy significantly improved the detection rate of CRC. However, its wide application is still limited due to high costs and inconvenience. Thus, specific sera-based tumor markers for early diagnosis and prognosis are needed.

Plasma/serum markers (e.g. Carcinoembryonic antigen, CEA) are the most widely used and reliable tumor markers for CRC because they are easily quantitatively measured, reproducible and cost effective. Although the current guidelines from the American Society of Clinical Oncology (ASCO) recommend that CEA levels should be monitored every 2–3 months for at least 2 years post diagnosis, there is no clear consensus on the validity of CEA monitoring [2]. Several studies [3]–[5] questioned the value of CEA as a marker of CRC recurrence, especially in patients with normal preoperative CEA levels. Due to these problems, new biomarkers are required to improve the early detection and prediction of recurrence for all kinds of CRC. Different proteomic patterns in serum present new opportunities to develop novel, highly sensitive diagnostic tools for early detection of cancers [6].

In the literature, proteomics technologies have been used to study CRC serum biomarkers. In these studies, proteomics strategies such as two-dimensional polyacrylamide gel electrophoresis (2-D PAGE), two-dimensional fluorescence differential in gel electrophoresis (2-D DIGE), surface-enhanced laser desorption/ionization time of flight mass spectrometry (SELDI-TOF MS) technology and protein arrays have been utilized,but none can precisely evaluate the level of up- or down-regulation of the proposed biomarkers. Therefore, it is difficult to determine their specificities and validity in blinded studies of the samples [7], [8]. To uncover new plasma biomarkers, a new approach with Isobaric Tags for Relative and Absolute Quantitation (iTRAQ) has been applied [9]. The stable incorporation of isotopes into an amine tagging reagent is involved in this chemical labeling method. By using mass spectrometry, proteins can be reliably detected and allow comparative quantitation in a multiplex manner. The core of this methodology is a multiplexed set of isobaric reagents that yield amine-derivatized peptides. The derivatized peptides are indistinguishable in MS, but exhibit intense low-mass MS/MS signature ions that support quantitation. This method imparts a mass difference as the basis for quantitation by measurement of relative peak areas of MS and/or MS/MS mass spectra. Today, commercially available 4-plex and 8-plex reagents can be used to label protein samples of interest following trypsin digestion. Different isobaric tags in this method suggest that in a single mass spectrometric analysis, up to 4 or 8 different samples, one of which is a reference, can be simultaneously examined. Given this reason, the iTRAQ approach is proposed as very promising in the discovery of biomarkers in samples with a wide range of plasma, body fluids and tissues.

In this study, iTRAQ was coupled with microQ-TOF/MS to detect differential expressed proteins in plasma from CRC patients and controls. Among the proteins that were elevated in CRC plasma, 9 proteins were up-regulated, and 4 were down-regulated. We discussed their possible functions and verified ORM2 which may be a potential serological biomarker for (the) CRC patients. The specific function of this protein has not yet been determined; however, ORM2 appears to function in modulating the activity of the immune system during the acute-phase reaction, which maybe play a potential role in the early development of CRC. Growing evidence indicates that tumor-associated inflammation can drive tumor development and progression, while tumor development and progression could induce inflammation, which may play a pivotal role in all stages of tumorigenesis. Such inflammatory molecules can be detected in blood samples from cancaer patients and may be have a potential role in the early detection of cancer. In this study, the up-regulation of ORM2 was confirmed by ELISA in plasma of patients with intestinal system disease.

## Results

### Identification of candidate biomarkers by microQ-TOF MS

For microQ-TOF MS protein profiling, plasma samples from 10 CRC patients and 10 individuals in the control group were collected (2 ml plasma from each individual) and then mixed to form a sample pool for further test. 100 µl of pooled samples from each group was immunodepleted of 12 abundant plasma proteins by immunoaffinity column technique as described in Methods.

Immunodepleted plasma samples were digested with trypsin, labeled with a unique isobaric tag, combined and simultaneously analyzed by microQ-TOF MS. CRC group was labeled with iTRAQ-114 and the healthy control group was labeled with iTRAQ-117 and then separated by strong cation exchange chromatography and C18 fractionation. 75 proteins were identified by microQ-TOF MS with ≥95% confidence. Among this group, 13 differentially expressed proteins were selected based on 1.5-fold over-expression and 1.5-fold under-expression in CRC patients, compared to the healthy volunteers. Nine proteins of the 13 differentially expressed proteins were up-regulated (Table 1). Three proteins were found to be significantly elevated in the CRC group (with ratios from 1.3 to 4.4). Four proteins were down-regulated in CRC patients, and their expression levels are shown in Table 2. The statistical variance of tumor versus normal ratios was within the 95th confidence level (P = 0.05).

**Table 1:** List of the proteins identified up-regulated in the iTRAQ experiments, which indicate the biological processes and molecular functions of these proteins (*P* value<0.001).

No.	Acc. number	Molecular Weight	Protein name	Ratio	Biological processes	Molecular functions
1	gi|153217289 (+7)	193 kDa	complement component 4a	0.6	complement-mediated immunity	complement component
2	gi|117911384 (+4)	44 kDa	unnamed protein product	1.3		
3	gi|112874 (+14)	48 kDa	alpha-1-antichymotrypsin precursor	4.4	lipid metabolism	other synthase
4	gi|112877 (+13)	24 kDa	alpha-1-acid glycoprotein 1 precursor	1	lipid metabolism	synthase
5	gi|119623346 (+10)	57 kDa	serpin peptidase inhibitor, clade d	0.7	serine protease inhibitor	proteolysis
6	gi|119607823 (+8)	24 kDa	orosomucoid 2	4.1	other miscellaneous function protein	immunity and defense
7	gi|223961	31 kDa	complement c4d	0.8	complement-mediated immunity	complement component
8	gi|3980130	72 kDa	unnamed protein product	1		
9	gi|15797461	19 kDa	unnamed protein product	0.7		

**Table 2:** List of the proteins identified as down regulated from the iTRAQ experiment, indicating is the biological process and molecular function of these proteins (*P*<0.001).

No.	Acc. number	Molecular weight	Protein name	Ratio (log2)	Biological processes	Molecular functions
1	gi|119570450 (+17)	23 kDa	retinol binding protein 4	−0.7	vitamin/cofactor transport	other transfer/carrier protein
2	gi|82492617	67 kDa	NADH dehydrogenase subunit 5	−0.7	oxidative phosphorylation	dehydrogenase
3	gi|114026 (+22)	11 kDa	apolipoprotein c-iii precursor	−0.7	regulation of lipid, fatty acid and steroid metabolism	other signaling molecule
4	gi|34532816	53 kDa	unnamed protein product	−1.6		

### Measurement of ORM2 in plasma samples

**Figure 1:**
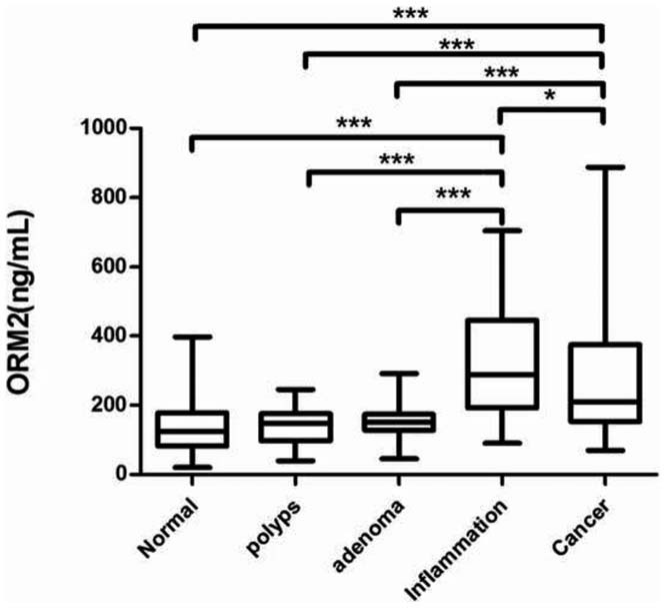
Serum ORM2 levels on a log scale in box-and-whisker plots. The star (stars) in the figure means a sign for statistical analysis. One star means P<0.05 and the sign with three stars mean P<0.001. CRC is statistically different from the normal colorectum, hyperplastic polyp and adenoma (all at P<0.001, respectively); IBD is statistically different from normal the colorectum hyperplastic polyp and adenoma (all at P<0.001, respectively). IBD is statistically different from colorectal cancer (P<0.05).

As shown in Fig. 1, plasma ORM2 levels on a log scale in box-and-whisker plots, median plasma ORM2 concentrations were significantly higher in CRC compared to the normal colorectum, hyperplastic polyp, and adenoma (all at P<0.001, respectively). The plasma ORM2 level was statistically significantly higher in patients with IBD than in the normal colorectum, colorectal hyperplastic polyp and adenoma (all at P<0.001, respectively) and it was statistically significantly higher in patients with IBD than in CRC (*P*<0.05).

We assessed whether plasma ORM2 could potentially distinguish CRC from benign colorectal diseases. We searched for correlation (age, gender, TNM stage and histological grade) between plasma ORM2 levels and CRC patient's clinicopathological parameters (Table 3). No significant association was found between plasma ORM2 concentrations and age, gender, TNM stage or histological grade.

**Table 3:** Clinicopathological characteristics of CRC patients.

Characteristic	No of specimens(N = 180)	Serum ORM2 levels(ng/mL)	*P* value
Gender			
Male	114	233.91 (156.22∼379.38)	0.3780
Female	66	200.62 (149.56∼339.68)	
Age (years)			
≤Median (68 years old)	93	219.04 (155.26∼378.73)	0.4567
>Median	87	207.57 (149.92∼361.37)	
TNM stages			
Stage I	49	188.86 (113.77∼375.23)	0.6211
Stage II	31	208.21 (179.28∼376.89)	
Stage III	62	232.90 (156.24∼363.52)	
Stage IV	38	208.43 (165.55∼405.93)	
Differentiated			
Well differentiated	61	235.82 (151.80∼434.52)	0.0761
Moderately differentiated	64	204.14 (150.69∼344.08)	
Poorly differentiated	55	219.03 (157.21∼343.07)	
Metastasis			
Yes	73	212.55 (157.93∼388.21)	0.3774
No	107	208.48 (152.56∼346.21)	

Values are Median (Inter-quartile range: IQR).

### Measurement of ORM2 in tissue samples

**Figure 2:**
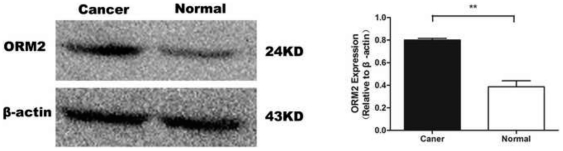
Expressions of ORM2 in CRC and corresponding adjacent normal mucous tissues by Western blot analysis (The sign with three stars mean P<0.01).

Western blot analysis was used to determine expression of ORM2 in tissues. When protein expression was measured by densitometer, the median densitometer value of ORM2 in CRC cancer tissues was significantly greater than that in corresponding adjacent normal mucous tissues (*P*<0.01) (Fig. 2).

## Discussion

In recent years, proteomics based biomarker discovery from serum/plasma has gained momentum. A number of studies have attempted to use proteomic approaches for the discovery of new biomarkers for CRC patients [10]–[14]. Studies by Yong-Sam Kim [10] revealed 26 candidate biomarkers for CRC by combined 2-DE and nano-LC-FT-ICR/LTQ MS. Ma et al [11] identified 4 proteins with diagnostic potentials by SELDI investigating the serum proteome of 62 CRC patients and 31 non-cancer subjects. However, issues regarding the complexity and dynamic range of constituent proteins have made it difficult to obtain meaningful data for interpretation. Both serum and plasma show tremendous variation in individual protein abundance. LC-MS/MS approaches for the analysis of serum/plasma samples afford much better dynamic range capabilities. In addition, iTRAQ reagents have gained interests in this field as a useful tool in protein biomarker discovery. It is the first study to combine iTRAQ with Q-TOF/MS on plasma specimens of CRC patients to discover potential biomarkers for CRC. 13 proteins with different expression patterns in CRC plasma were successfully identified. Among these 13 proteins, there were several proteins of interest, such as ORM2, Serpin peptidase inhibitor (clade D), NADH dehydrogenase subunit 5 (ND5) and Retinol binding protein 4 (RBP4), which were selected as beneficial candidates and may be further investigated. ORM2 (alpha-1-acid glycoprotein), a member of the acute-phase protein family found to be related to intestinal system disease [15]–[18], was selected for further verification and investigation.

It is known that cancers are accompanied by numerous systemic physiological and biochemical changes, including glycosylation which is one of the most important post-translational modifications of proteins. More and more attentions are being paid on studying aberrant glycosylation during various pathophysiological states, including inflammation [19], arthritis [20], [21], and cancer [22], [23]. During the organization of our data, we found several serological glycosylated proteins like ORM2, EpCAM changing in levels during cancer development. In this preliminary research, we reported ORM2 as a new member of these cancer-associated glycoproteins. Wandall [24] used a novel O-Glycopeptide microarray to screen seromic profiling of patients with colorectal cancer.Dai [25] individually imaged the change in glycoproteins indirectly. These reports encourage another angle to discover clinical biomarkers for diagnosis and prognosis.

There are several reports of ORM2 in intestinal system disease and cancers [15]–[18], [26]. ORM2 was considered to be related to capillary permeability [15]. The function of orosomucoid was reported to be linked with inflammation and cancer. The concentration of S-orosomucoid was found to be increased in patients with Crohn's disease [16]. Comparing bladder cancer patients with healthy controls, there were significant differences, with much more orosomucoid expressed in the cancer group [17]. Similar phenomenon could be also found in patients with bronchial carcinoma [18]. Using the 2-D DIGE and further analyzed by MALDI-TOF mass spectrometry in patients with chronic inflammatory demyelinating polyneuropathy, significant up-regulation of alpha-1 acid glycoprotein 1 precursor was identified [26]. Studies have indicated that CEA, similar to alpha 1-acid glycoprotein (orosomucoid) in immunology is postulated as a biosynthetic precursor of alpha 1-acid glycoprotein [27].

This is the first report of an up-regulation of ORM2 in CRC plasma. Furthermore, the up-regulation of ORM2 was confirmed by ELISA in plasma of patients with intestinal system disease. It was found that ORM2 were significantly increased in patients suffering from CRC and IBD compared with normal individuals and patients with hyperplastic polyp and adenoma. ORM 2 is a key acute phase plasma protein in inflammation, so this protein is classified as an acute-phase reactant. The specific function of this protein has not yet been determined, however, ORM2 appears to function in modulating the activity of the immune system during the acute-phase reaction. This may serve as a potential mediator of immune escape in CRC and the detection of ORM2 may be significant in distinguishing colorectal carcinogenesis from IBD.

To determine the source of ORM2, the CRC tumor tissue and corresponding adjacent normal mucous tissues were evaluated by Western blot. The results showed that expression level of ORM2 in CRC cancer tissues was found to be higher than in normal tissue. Therefore, it is proposed that ORM2 might be involved in carcinogenesis in patients with CRC cancer.

This study also provides evidence that ORM2 levels in CRC patients may be useful as a biomarker for diagnosis. Serum CEA assays are not recommended for screening asymptomatic patients for cancer. One reason is that the plasma CEA assay is not diagnostic enough to discriminate between localized malignant tumors and benign disorders. In our study, the detection of ORM2 using ELISA and Western blot is promising. Plasma ORM2 concentrations were significantly higher in CRC compared to the normal colorectum, hyperplastic polyp, and adenoma (P<0.001). Therefore, using ORM2 to monitor potential CRC patients may be a good way to validate whether ORM2 can be used for screening asymptomatic patients. However, it is important to further analyze the diagnostic significance of ORM2 by a large number of patients in multiple centers before final clinical application for CRC diagnosis.

Serpin peptidase inhibitor, clade D (heparin cofactor), the encoded product heparin cofactor which shares homology with antithrombin III and other members of the alpha 1-antitrypsin superfamily can rapidly inhibit thrombin in the presence of heparin. Defects in SERPIND1 are the main cause of heparin cofactor II deficiency, and heparin cofactor II deficiency can lead to increased thrombin generation and an a-hypercoagulable state. This is disadvantageous to the development of cancer. There are several reports on clade A and E in cancers [28], [29], but few on clade D. Similar protein was also identified as an Alpha-1-antichymotrypsin precursor. Alpha-1-antichymotrypsin belongs to the serpin family, is highly expressed in astrocytes and has been found to play roles in the pathogenesis of classical plaques with Alzheimer's diseases. As an acute-phase factor, it can inhibit the activity of macrophage ectoenzymes and matrix metalloproteinase-9 during skin wound healing [30], but induces TNF-α production in the study of microglial cell lines [31]. This type of protease inhibitor has been reported to form complexes with the kallikrein family such as hK3 (also known as a prostate-specific antigen), hK2 and hK6, and could be applied in the diagnosis of prostate cancers [32].

Among the down-regulated proteins, ND5, an essential subunit component of whole mitochondrial complex I [33], [34], plays an important role in the oxidative phosphorylation process as well as in carcinogenesis. Some literature indicated that ND5 could induce hepatomas [35]. Mitochondrial DNA (mtDNA) mutations have been reported in many kinds of cancers. mtDNA gene defects may be involved in the pathogenetic mechanisms of fatal manifestation [36]. Frame-shift mutation in ND5 genes has been reported in hepatocellular carcinoma, and also in preneoplastic lesions of the gastrointestinal tract [37]. In this study, we identified ND5 (67 kDa) as a potential candidate but its precise role in carcinogenesis still needs further investigation, especially the mutations.

RBP4, a cytokine secreted by adipose tissue, is a novel adipokine linked with obesity and insulin resistance of type 2 diabetes. RBP4 also has been demonstrated to be involved, to some extent, in the development of inflammation and cancer. For example, RBP4 methylation was investigated in esophageal squamous cell carcinoma [38]. In colorectal carcinoma, the level of RBP decreases [39]. In our study, we identified RBP4 as a prospective candidate but further investigation of its function in the pathogenesis of cancer development is required.

In this preliminary proof-of-principle study, Table 1 and Table 2 list 13 candidate biomarkers for CRC. Most of these have not been reported as cancer biomarkers, that have shown that iTRAQ together with Q-TOF/MS is a sensitive, reproducible, robust technique for identifying statistically significant differences of protein expression betweenCRC patients and healthy control plasma samples. From the verification of ORM2, it was found that ORM2 may be a potential biomarker to distinguish CRC from IBD. In future studies, larger sample populations will be required to confirm these potential biomarkers. In our research, ORM2 is firstly identified as a differently regulated protein in CRC patients as compared to normal controls with proteomics screening. However, it requires a lot of bench experiments and clinical tests before ORM2 can be used as a reliable biomarker. We will continue our study on ORM2 in two aspects. Firstly, we are establishing a platform to distinguish and to analyze the relevancies of distinct levels on glycosyl pattern modifications to various phases of diseases development based on ORM2. At the same time, we are also very interested in the function of ORM2 in the tumour progression owing to it regulates lipid homeostasis and protein quality control on the endoplasmic reticulum membrane.

## Methods

### Sample preparation

This study was approved and monitored by the Ethics Committee of Qilu Hospital, Shangdong University. Plasma samples for proteomics from 10 CRC patients group and 10 healthy control group were collected (Table 4). For each person, 2 ml blood was obtained by venipuncture and collected in a tube with EDTA, and the plasma was prepared as recommended by the HUPO Plasma Proteome Project [40]. There were a total of 419 subjects for ELISA, which were classified into five categories by standard clinical, radiological endoscopic and histological criteria: normal control (n = 65), hyperplastic polyp (n = 59), inflammatory bowel disease (IBD) (n = 62), adenoma (n = 53) and CRC (n = 180). For Western blot, a total of 41 sequential patients diagnosed as CRC were assessed. Tumor specimens and corresponding adjacent normal mucous tissues (>5 cm from the margin of the tumor) were collected and stored at −80°C until use. No patients in this studyreceived any type of therapy, such as radiation or chemotherapy.

**Table 4:** Clinical information of CRC serum specimens.

Sample number (internal code)	Age (years)	Gender (male/female)	Differentiation	Histological types	TNM/DukesState, Stage	Preoperative CEA (ng/ml)
1	54	female	moderate	adenocarcinoma	T_3_N_0_M_0_/B, II	2.5
2	56	female	moderate	adenocarcinoma	T_3_N_0_M_0_/B, II	3.1
3	45	male	moderate	adenocarcinoma	T_3_N_0_M_0_/B, II	4.6
4	63	male	poor	adenocarcinoma	T_4_N_1_M_0_/C, III	3.7
5	51	male	poor	adenocarcinoma	T_3_N_1_M_0_/C, III	4.2
6	69	female	poor	adenocarcinoma	T_3_N_0_M_0_/B, II	2.9
7	55	female	moderate	adenocarcinoma	T_2_N_0_M_0_/A, I	3.8
8	65	male	moderate	adenocarcinoma	T_2_N_0_M_0_/A, I	4.8
9	47	female	poor	adenocarcinoma	T_3_N_1_M_0_/C, III	5.3
10	33	male	poor	adenocarcinoma	T_4_NM_1_/D, IV	5.7

TNM: tumor-node-metastasis classification system.

Pathological type: ulcerative type/protruded type/infiltrating type.

Lymph node metastasis: yes/no.

Depth of invasion: T1/2/3/4.

### Immunodepletion of high-abundance plasma proteins

Plasma samples from 10 CRC patients and 10 individuals in the control group were collected (2 ml plasma from each individual) and then mixed to form a sample pool for further test. Pooled plasma samples were depleted of the top 12 most abundant proteins (Albumin, IgG, IgA, IgM, Transferrin, Fibrinogen, α_2_-Macroglobulin, α_1_-Antitrypsin, Haptoglobin, α_1_-Acid Glycoprotein, Apolipoprotein A-I, and Apolipoprotein A-II) using a pre-packed 2-ml Seppro™ MIXED12 affinity LC column (GenWay Biotech, San Diego, CA) on an Agilent 1100 series HPLC system (Agilent, Palo Alto, CA) according to the recommended manufacturer's procedure.

After depletion, the precipitated proteins were dissolved by lysis buffer (6 M Urea, 4%CHAPS, 1 mM PMSF, 2 mM EDTA). Protein concentrations were determined by the 2D Quant Kit (GE healthcare, USA), using albumin as a reference standard (1 µg/µl–10 µg/µl). Concentrations were measured to 4.83 µg/µl (the CRC group) and 2.27 µg/µl (the healthy group).

### Trypic digeston and iTRAQ reagent labeling

After reduction (10 mM DTT, 100 mM NH4HCO3, 56°C, 45 min) and alkylation (55 mM IAA, 100 mM NH4HCO3, 45 min), each sample (100 µg) was digested with trypsin and labeled with iTRAQ reagents. These samples were digested in dissociation buffer (trypsin∶protein = 1∶30) overnight at 37°C. Peptides were labeled with iTRAQ reagents (114 for the CRC group, 117 for the healthy group).

### Strong cation exchange chromatography and C18 column fractionation

Strong cation exchange chromatography was used to remove the redundant iTRAQ reagents and interfering substances that can affect MS analysis. The labeled samples were added to 10× Buffer A (10 mM KH2PO4 in 25% acetonitrile, pH 3.0). High-resolution cation exchange chromatography was then performed to separate the labeled samples into 10 fractions. Peptides were eluted with a linear gradient of 0∼500 mm KCl (25% v/v ACN, 10 mm KH2PO4, pH 3) over 15 min at a flow rate of 1 ml/min, with fractions collected at 1-min intervals. Samples were desalted and further fractionated by using a C18 column on nano HPLC (PROXEOME, America) followed by MS analysis. The gradient was 5∼40% ACN for 70 min.

### Matrix assisted laser desorption ionization-time of flight (MALDI-TOF)/TOF and microQ-TOF analysis

Machine parameters were set as, ion source 25 kV for positive matrix factorization (PMF) and 8.0 kV for tandem MS/MS reflector 26.3 kV for PMF and 29.5 kV for tandem MS/MS, lens 9.5 kV for PMF and 3.5 kV for tandem MS/MS, lift 19.0 kV and the spectra were acquired in reflection mode in MALDI TOF/TOF with mass range 700∼4000. The peaks with a ratio of S/N over 5 were automatically labeled by FlexAnalysis (Bruker, Germany). The PMF peaks with mass intensity of more than 5000 were selected for further MS/MS. The spectra were acquired in positive ion mode in microQ-TOF with mass range 50∼3000, and the dry gas and the nebulizer gas were set as 3 L/min, and 2 L/min, respectively. The temperature of the spray chamber was set as 150°C, and the strength of the electric field was set as 1400 V, the collision energy was set as 35% timing for low ion mass and 65% timing for high ion mass. Other parameters were set as default.

### Database search

The spectra were processed with microQ-TOF control 3.0 (Bruker, Germany) using default settings, except that the parameters of transfer funnel RF and collision cell RF were set as 120 Vpp and 600 Vpp, respectively. The subsequent data analysis was carried out using the Data Analysis 4.0 Biotools 3.0 (Bruker, Germany), WARP-LC 2.0 (Bruker, Germany), and Mascot 2.2 (Matrix Science, London, U.K.), respectively. Protein identification was performed by searching the Swiss-Prot Human2009–12 database with a precursor ion and fragment mass tolerance both set as 0.04 Da. Carbamidomethylation of cysteines was set as fixed modification, and oxidation of methionines, iTRAQ 8 plex modification of N terminal, K and Y were considered as variable modifications, respectively. One maximum mis-cleavage was accepted. The ion score cutoffs were set to 25 for peptides and to 68 for proteins. Peptide identifications were accepted if they could be established at greater than 90.0% probability as specified by the PeptideProphet algorithm. Protein identifications were accepted if they could be established at greater than 90.0% probability or contained at least 2 identified peptides. Proteins that contained similar peptides and could not be differentiated based on MS/MS analysis alone were grouped to satisfy the principles of parsimony. For classification, the gene ontology (GO) symbols of the identified proteins were curated using the XRef database, and queried against the gene ontology database using the GoMiner tool (http://discover.nci.nih.gov/gominer/index.jsp). Prediction of the origin of proteins with unknown cellular localization was performed using the PSORT II (http://www.psort.org/).

All protein iTRAQ ratios were transformed to base 2 logarithm values. In base 2 logarithm space, a 2-fold change in levels was reported as 1 or -1 for up-regulated and down-regulated changes, respectively.

### Elisa for ORM2

ORM2 levels in plasma were quantified using a commercially available ELISA kit (Groundwork Biotechnology Diagnosticate, San Diego, CA), according to the manufacturer's instructions.

### Western blotting for ORM2

Tumor tissues and corresponding adjacent normal mucous tissues were re-suspended in ice-cold radioimmunoprecipitation (RIPA) buffer for lysis. A BCA protein assay kit (Beyotime, Biotechnology, China) was used for protein assay. About 25 µg protein of samples was loaded, separated on 9.0% Tris-Glycine-SDS polyacrylamide gels and then electro-transferred onto polyvinylidene fluoride membranes. After blocking with 5% non-fat milk, membranes were incubated with ORM2 Ab (1∶150, Yueyan Biological Technology, Shanghai, China) or anti-β-actin mouse monoclonal antibody (1∶500, Santa Cruz Biotechnology, Santa Cruz, CA) overnight at 4°C. Membranes were further incubated with polyclonal mouse anti-Goat HRP-conjugated secondary antibody (Santa Cruz Biotechnology, Santa Cruz, CA) or polyclonal goat anti-mouse HRP-conjugated secondary antibody (Santa Cruz Biotechnology, Santa Cruz, CA) for 2 h at room temperature. Bands were detected using a chemiluminescence detection kit (Amersham Pharmacia Biotech, Piscataway, NJ). The intensity of each band was quantified using ImageJ 1.32 software (National Institutes of Health, Bethesda, MD) after densitometric scanning of the films.

### Statistical analysis

The Kolmogorov–Smirnov test was performed to determine the distribution of the samples of each group. Data were expressed as median (inter-quartile range, 1IQR). A non-parametric test (Kruskal–Wallis test) was applied to analyze differences between CRC and other groups. P<0.05 was considered as statistically significant. Statistical analysis was performed with SPSS software versions 13.0 for windows (SPSS Inc, Chicago, USA).
